# Pore Structure, Morphology, and Strength of Self-Compacting Foam Material Backfilled Behind the Underground Pipe-Wall of Yellow River

**DOI:** 10.3390/ma13245724

**Published:** 2020-12-15

**Authors:** Jian Zhao, Yansong Deng, Peiwei Gao, Xiaolin Lu, Jun Zhang, Jianjun Zong

**Affiliations:** 1 Department of Civil Engineering, Nanjing University of Aeronautics and Astronautics, Nanjing 210016, China; zhao1062020@163.com (J.Z.); lxl_msc@nuaa.edu.cn (X.L.); zhangjun_afeu@sina.com (J.Z.); zjj199908@163.com (J.Z.); 2 Jiangxi Changtong Highway Co. LTD, Nanchang 330025, China; dengyansong2020@163.com

**Keywords:** self-compacting foam material, pores structure, compressive strength, backfilled underground pipe wall, Yellow River embankment

## Abstract

The backfill material for the underground pipe wall of Yellow River embankment was developed to avoid the high settlement and environmental damage caused by high filling and excavation. The interrelation between microstructure and compressive strength of the self-compacting foam backfill material with different water–binder ratios and density grades was investigated. The results indicated that the average pores size of the foam backfill material increased with increasing the water–binder ratio. Moreover, the compressive strength of the foam backfill material first increased and then decreased with continuously increasing the water–binder ratio. Based on the observation and the analysis of the computed tomography (CT) image, it can be found that the pore size of the foam backfill material decreased with the increment of the density grade. The overall findings demonstrated that the pore size and volume played an important role in affecting the performance of the foam backfill material used for the construction of the underground pipe wall of Yellow River embankment.

## 1. Introduction 

The Yellow River is an important waterway connecting the inland to the coast of China. Its dike is a momentous part of the national flood control system. In recent years, the number of water or gas tunnel and subway tunnel projects has been increased [1,2]. These tunnels are inevitable to cross the Yellow River and Yangtze River from underwater. A large number of culverts and highways have been built on the embankment. Distinct from the bridges structures or other river embankments, the foundation of the Yellow River embankment is mostly silt, silty clay, clay, medium sand, etc. It has low soil cohesion, poor foundation conditions, and built nearby the river. The selection of the embankment district line is often limited by the trend of the river, and most of the dike foundation has not been subject to foundation treatment. 

Most of the researches show that the increment of the dike projects through the Yellow River embankment would reduce the strength of the soil. When the soil in the embankment encounters rainfall, the soil strength decreases due to the increment of water content, which might cause the slide and collapse at the top of the embankment. In the construction and operation period, the penetration of pipeline might affect the safety of the dike. If the construction and backfill areas surrounding the pipelines or tunnels are not compact, the settlement, deformation, seepage, and displacement of the soil around the tunnels will occur [3,4]. This will exert a significant impact on river flooding, river stability, and embankment safety. 

The self-compacting foam lightweight material is an attractive construction material in the production of backfilled underground pipe wall. It holds several advantages over traditional materials, such as self-compacting property, and ultra-low self-weight [5,6,7]. Besides, it possesses excellent structural properties, such as low permeability and water absorption, excellent insulation performance, anti-freezing, and independent application after curing [8,9,10]. At the same time, compared with ordinary concrete, foam lightweight material has a certain reduction effect on structural members under the excitation of seismic force due to its ultra-low self-weight. As a result, the internal force and displacement of the structure are significantly reduced, effectively improving the shear bearing capacity and deformation characteristics of the structure [11,12]. These properties make the lightweight foam materials suitable for applications in filling the back-wall of the Yellow River pipeline to avoid high settlement and environmental damages caused by high filling and digging, which is of great significance for protecting the dike safety and natural ecological environment [13,14,15]. The Shanxi Yellow River Diversion Project employed the foam concrete with a density of 1200 kg/m^3^ in the coupling section of the concrete structures. The employed foam concrete could fill the gap between the tunnels and fulfill the design requirements for compressive strength and construction pouring [2].

Currently, the backfilling method could be conducted by grouting raw soil materials. However, only applying backfilling once would not result in a dense structure. Thus, repeated backfilling is normally needed. Moreover, because of the long installation time, a prolonged construction period might affect the settlement and lead to the collapse of the embankment [16,17]. These issues tend to cause critical accidents, such as the collapse of the dikes and adjacent buildings. During the construction of the Large Yellow River tunnel or water pipeline, there are a large number of backfilling areas behind the pipeline wall. The construction treatment of these areas not only needs to fulfill the mechanical properties but also requires to make these areas compacted with a low settlement and finished within a short time. Therefore, the backfill materials should have a high fluidity, volume stability, and short setting time. To meet these requirements, the stabilized and admixture materials are added in the lightweight materials to improve their strength, fluidity, shrinkage settlement performance, and sustainable development [18,19].

The self-compacting foam lightweight material has been used as a backfill material for a long time in a subterranean and humid environment, where high comprehensive performance in all aspects of the substrate is needed [20,21]. This study employed a high-performance foaming agent XM-1 to prepare the self-compacting foamed lightweight filling material. A wide range of variations in water–binder ratio and fly ash content were investigated to optimize the mixed design of the developed material. Then, the property of the self-compacting foam lightweight backfill material were examined. Moreover, the pore structures and parameters of the substrate were studied using CT scanning. The influencing mechanisms of macro-factors in the microstructure were dissected to provide a basic theory for the backfill application of the developed self-compacting foamed lightweight material in the underground pipeline wall of the Yellow River embankment.

## 2. Experiment Materials and Methods

### 2.1. Experiment Materials

According to the requirements of Chinese standards, the 42.5 MPa Portland cement, class II of fly ash, foaming agent, and water reducer are used to prepare the experimental specimens. The FC-1 foaming agent developed by our research group is employed, which is diluted to the targeted concentration (one fortieth of its original concentration) prior to use. The nature of the foaming agent significantly affects the properties of foamed concrete [22]. The characteristics regarding the foaming agent used in the experimental campaign are shown in Table 1. Polycarboxylic acid is employed as the water reducer with a water reduction rate of about 32%. The properties and index of the natural river sand and clay soil are shown in Table 2.

### 2.2. Foam Backfill Material Preparation Procedures

#### 2.2.1. Preparation of Foam

The pre-foaming method is used to prepare the foam added in the foam lightweight material. The prepared foaming agent stock solution is diluted with water in a certain proportion, and then the foaming liquid is foamed by the MC-600 high-pressure air foaming machine (Hebei, China).

#### 2.2.2. Preparation of Foam Backfill Material

The weighed base materials are added to the concrete mixer (a forced-type concrete single-shaft mixer) (Zhengzhou, China) and are mixed uniformly for 2 min at a rotation speed of 45 rpm. Then, the foam is added into the slurry, and the mixture is mixed for another 2 min until the foam and cement-based materials are completely fused. 

### 2.3. Test Methods and Mix Ratios of Foam Backfill Material

In the mixture design of the foam backfill material, the amounts of raw materials for different water–binder ratios are calculated based on the total mass of foam backfill material per unit volume, and the mix ratios are shown in Table 3.

The water to binder ratio (W/B) is the ratio of the amount of water to the amount of cementitious material. The compressive strength, porosity, and average pores size tests of the substrate suffice the requirements of Foam Concrete (JG/T266-2011) and Technical Regulations for Filling Lightweight Soil Filling Project (CJJ/T 177-2012).

In the mix design of foam backfill material, the density of the foam backfill material is mainly determined by the amount of used foam. Herein, the volumetric method is employed to design the mix ratio (the base material per unit volume). Considering that the foam will be destroyed during the mixing process, the sum of the substrate volume plus the foam volume is applied as 1 m^3^, as shown in Equation (1).

(1) $$V_{c}+V_{w}+V_{s}+\frac{V_{f}}{Q}=1m^{3}$$

Equation (1) can also be written as:

(2) $$\frac{M_{c}}{\rho_{c}}+\frac{M_{w}}{\rho_{w}}+\frac{M_{s}}{\rho_{s}}+\frac{M_{f}}{\rho_{f}Q}=1m^{3}$$

where, *V*, *M*, and *ρ* represent the volume, mass, and density of the material, respectively. The sub-symbols of *c*, *w*, *s*, *f* represent cement, water, filler, and foam, respectively. *Q* is the experience of damage coefficient.

#### 2.3.1. Dry Density of Backfill Foam Materials

The test method for the dry density of foam backfill materials is based on the Chinese standard JC/T266-2011. Three of the specimens are taken as one group. The length in different directions for each specimen is measured, and the average value (V) of the volume for each specimen group is calculated.

Each specimen is placed in an oven (60 ± 5) °C for 4 h until the mass difference between the two specimens weighted immediately after each other is less than 1 g. The specimens are then cooled to room temperature in a desiccator and weighed to calculate the average mass of each specimen. The equation of dry density is denoted as Equation (3):

(3) $$\rho_{0}=\frac{m_{0}}{V_{0}}\times 10^{6}$$

#### 2.3.2. Compressive Strength of Foam Backfill Materials

The average compressive strength value of three specimens is calculated and taken as one group, and the measurement accuracy of each group is ±1%. The machine press employed in the test complies with the corresponding technical requirements of Chinese standard GB/T2611-2007. First, the force from the bottom area of the specimen is measured and calculated. Then, the load is applied continuously at a constant speed until the specimen is fractured. The maximum load is recorded, and the compressive strength is determined. 

The completely mixed mortar is cast in a 100 × 100 × 100 mm^3^ test mold and then vibrated to obtain a uniform mixture. After 24 ± 2 h, the mold is removed, and the specimens are placed in a standard curing box for 28 days. The compressive strength and pores structure are then measured. The specimens of the foam backfill material at 28 days of curing are cut into pieces. Their surface images are observed, and the size of the average pores of the foam backfill material is calculated. The porosity of the foam backfill material measured by the mass volume method is calculated by Equation (4).

Porosity = *(ρ* − *ρ*_0_)/*ρ* (4)

where, *ρ* and *ρ*_0_ are the dry density of the specimen (kg/m^3^) and the actual density of the specimen (kg/m^3^), respectively. 

**Figure 1:**
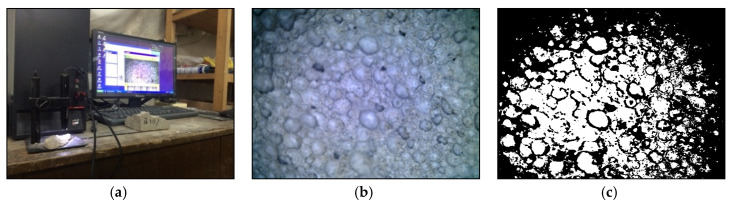
Image processing of porosity, (**a**) image acquisition, (**b**) original image of porosity, (**c**) grayscale porosity.

The test piece is cut into a smooth section and placed under a supereyes microscope (produced by a technology company in Shenzhen) to extract an image with a suitable focal length. The grayscale image is obtained hereafter by the gradation processing of these collected aperture images. As shown in Figure 1 (the picture is a microscopic view of the microscopes at a magnification of 200), the unit of the grayscale image is converted to mm, and the area is calculated to obtain an average aperture.

## 3. Results and Discussion

### 3.1. Average Pores Size and Porosity of the Foam Backfill Material

**Figure 2:**
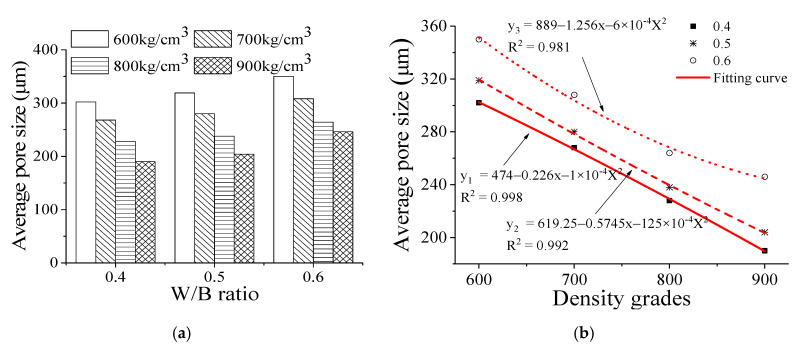
Effect of water–binder ratio on average pore size of the foam backfill material at different density grades. (**a**) W/B ratio; (**b**) Density grades.

Figure 2 shows the average pores size and porosity of the foam backfill material with different density grades and water–binder (*W/B*) ratios. The average pore size of the foam backfill material with different density grades is increased with increasing water–binder ratio. For example, the average pore size of the foam backfill material with a water–binder ratio of 0.50 and density grades of 600–900 is increased by 5.6%, 4.5%, 4.4%, and 7.4%, respectively, compared with those with a water–binder ratio of 0.40. The corresponding average pore size of the foam backfill material with a water–binder ratio of 0.60 from grades 600–900 is further increased by 9.7%, 10%, 10.9%, and 20.6%, respectively. Apparently, the increment of the average pore size for the water–binder ratio from 0.40 to 0.50 is lower than that for the water–binder ratio from 0.50 to 0.60. These results are also significantly performed on the regression map (Figure 2b). It can be seen that the R^2^ of the W/B ratio of 0.40 and 0.50 is higher than that of 0.60. Their significant values are 0.998, 0.992, and 0.981, respectively.

As the density grade of the foam backfill material increased, their average pore size decreased correspondingly. For example, when the density grade increased from 600 to 900, the average pore size of the foam backfill material with a water–binder ratio of 0.40 is 302 μm, 268 μm, 228 μm, and 190 μm, respectively, which are reduced by 11.3%, 24.5%, and 37.1%, respectively. Within the same density grade range, the average pore size of the foam backfill material with a water–binder ratio of 0.60 is 350 μm, 308 μm, 264 μm, and 246 μm, which are reduced by 12.0%, 24.6%, and 29.7%, respectively. It can be concluded that in the density grade range of 600 to 900, the larger the water–binder ratio of the foam backfill material, the larger the reduction of the average pores size.

**Figure 3:**
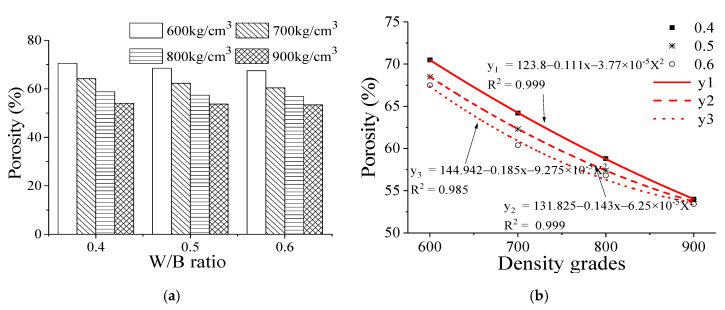
Effect of water-to-binder ratio on porosity of the foam backfill material at different density grades. (**a**) W/B ratio; (**b**) density grades.

Figure 3 shows that when the water–binder ratio is increased, the porosity of the backfill foam material in different density grades is reduced slightly. For instance, in grade 600, the porosity of the foam backfill material with the water–binder ratio of 0.40, 0.50, and 0.60 is 70.5 kg/cm^3^, 68.5 kg/cm^3^, and 67.5 kg/cm^3^, respectively, which are respectively decreased by 2.8% and 4.3%. At the same water–binder ratio, the porosity of the foam backfill material is decreased with increasing the density grade from 600 to 900. For instance, at a water–binder ratio of 0.4, the porosity of the foam backfill material is decreased by 8.9%, 16.6%, and 23.4%, respectively, with increasing the density grade from 600 to 900. 

As the water–binder ratio increases, the bonding properties of cementitious materials decrease gradually, and their ability to encapsulate air bubbles weakens correspondingly. This may be because as the water-to-binder ratio increases, the cohesive force of the slurry decreases. As a result, a large number of bubbles undergo transition and fusion. During the fusion process, small bubbles burst, causing a reduction in the overall porosity. Consequently, a large number of air bubbles are interconnected to form larger bubbles, which in turn increases the average pore size of the backfill foam material, particularly when the water–binder ratio is increased from 0.50 to 0.60. If the water binder ratio of the foam backfill material is further increased, the size of the air bubbles will be limited by the adhesive stress between cementitious materials, and the growth rate of the pore size will slow down correspondingly [23,24,25]. At the same water–cement ratio, the average pore size of the foam backfill material with different densities decreases with the increase of the density grade. It can be expected that the proportion of the cementitious materials would be increased along with the increment of the density grade of the foam backfill material. During the formation and movement of the air bubbles, the resistant force encountered by the pores increases, resulting in a decrease in the average pore size. This phenomenon shows significant performing in the regression map (Figure 3b), in which, the R^2^ of the W/B ratio of 0.40 and 0.50 is higher than that of 0.60. Their significant values are 0.999, 0.999, and 0.985, respectively. 

### 3.2. The Compressive Strength of the Foam Backfill Material

**Figure 4:**
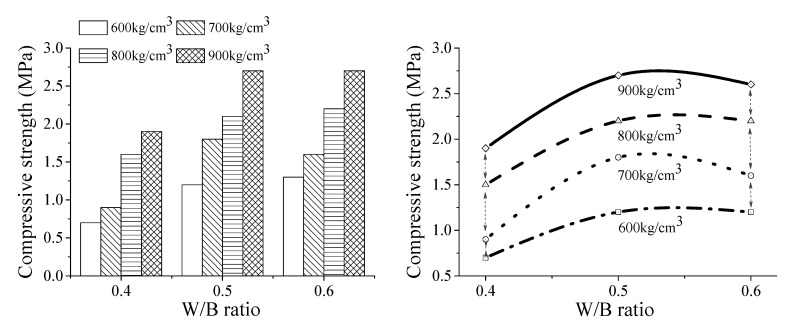
Effect of water-to-binder ratio on the compressive strength of the foam backfill material at different density grades.

The compressive strength of the foam backfill material in different density grades as a function of the water–binder ratio is shown in Figure 4. At a fixed water–binder ratio, the compressive strength of the foam backfill material increases gradually with increasing density grades. For instance, at a water–binder ratio of 0.40, the compressive strengths of the foam backfill materials from density grades of 600 to 900 are 0.7 MPa, 0.9 MPa, 1.5 MPa, and 1.95 MPa, respectively, which are respectively increase by 28.6%, 114.3%, and 178.6%.

**Figure 5:**
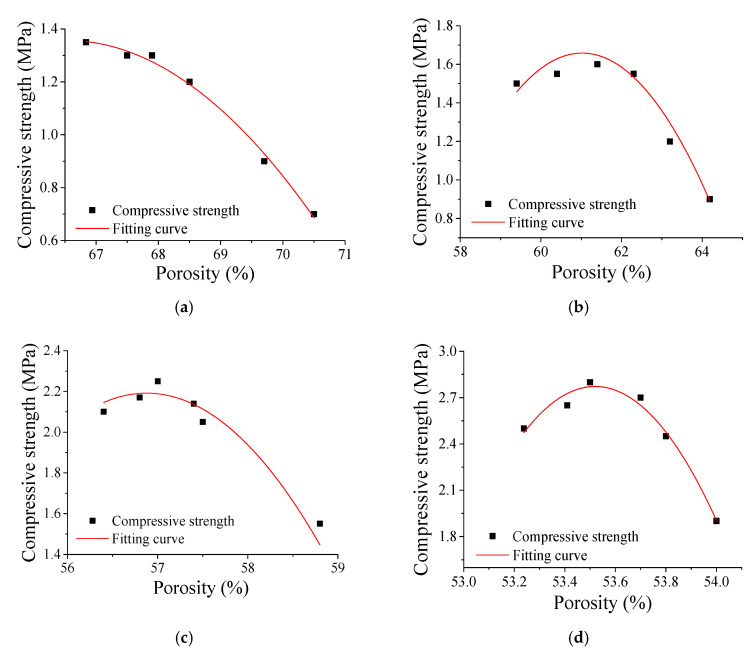
Correlation between porosity and compressive strength. (**a**) 600 kg/cm^3^ foamed concrete; (**b**) 700 kg/cm^3^ foamed concrete; (**c**) 800 kg/cm^3^ foamed concrete; (**d**) 900 kg/cm^3^ foamed concrete.

At a fixed density grade, the compressive strength of the foam backfill material first increases and then decreases with increasing the water–binder ratio continuously. The regular pattern of variation is shown in Figure 5, and their fitted parameters of the regression equation are shown in Table 4. With a density grade of 900, the compressive strengths of the foam backfill materials with a water–binder ratio of 0.4, 0.5, and 0.6 are 1.95 MPa, 2.7 MPa, and 2.68 MPa, respectively, which are increased successively by 38.5% and 37.4%, respectively.

The cohesiveness of cementitious materials in the foam backfill material decreases with increasing the water–binder ratio, which encourages the integration of air bubbles and reduces their total volume in the matrix. The high flexibility of the paste allows cementitious materials in the foam backfill material to hydrate more completely. As a result, more hydration products fill the voids of the paste, effectively reducing the porosity and thereby increasing the strength of the backfill foam material. However, further increasing the water–binder ratio decreases the porosity but increases the pore size, resulting in the uneven distribution of pores in the foam backfill material and consequently reducing its compressive strength. 

### 3.3. Morphologies of the Foam Backfill Material with Different Density

**Figure 6:**
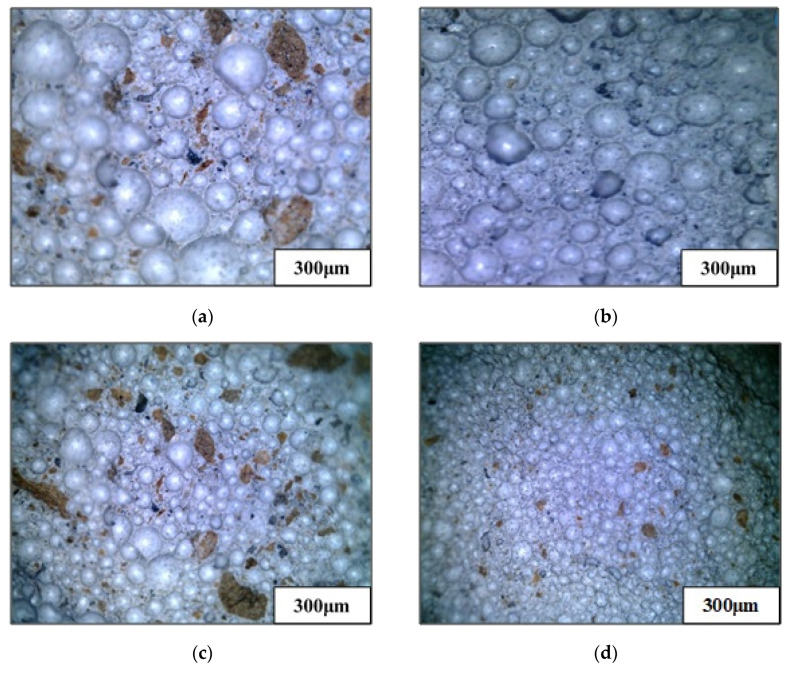
The topography of the foam backfill material with different densities at a 0.5 W/B ratio. (**a**) 600 kg/cm^3^ foamed concrete; (**b**) 700 kg/cm^3^ foamed concrete; (**c**) 800 kg/cm^3^ foamed concrete; (**d**) 900 kg/cm^3^ foamed concrete.

Figure 6 shows the topography of the foam backfill material incorporated mixtures with water–binder ratio of 0.5 and different densities. It can be seen that when the density grade increases, the diameter of the air bubbles in the foam backfill material decreases correspondingly. For instance, the pore size of the foam backfill material with the density grade of 600 kg/m^3^ is significantly larger than that of the other grades, as shown in Figure 6a. These results are consistent with the average pore size test results in Figure 1. 

Meanwhile, as the density grade increases, the amount of air bubbles increases significantly, the distribution of pores becomes more uniform, and the space between the air bubbles narrows down, and the diameter of the air bubbles decreases. All these changes in the microstructure are consistent with the porosity test results in Figure 5. The density of the foam backfill material increases with a decrease in the porosity. The contact surface and adhesiveness of cementitious materials are increased, which indirectly promotes the cement hydration reaction and thereby improving their compressive strength. These observations correspond well with the compressive strength of the foam backfill material in Figure 3.

**Figure 7:**
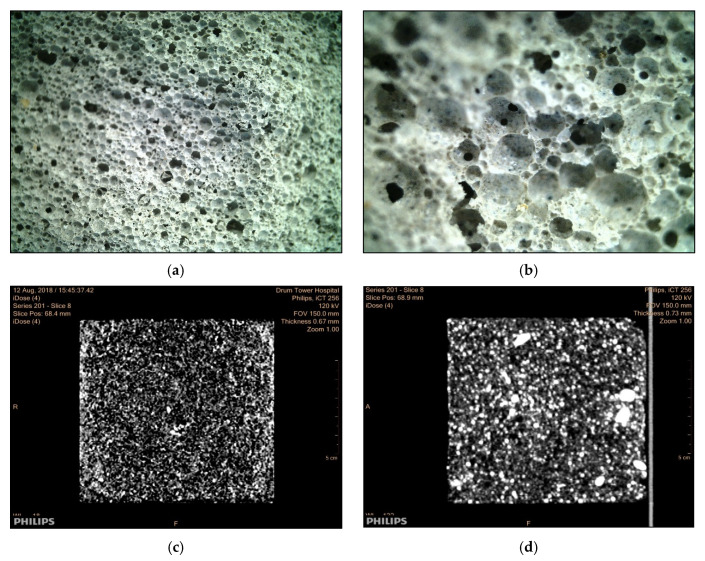
Pore morphology of the foam backfill material with different *W/B* ratios. (**a**) 0.40 *W/B* ratio, (**b**) 0.50 *W/B* ratio, (**c**) 0.40 *W/B* ratio through the CT scan, (**d**) 0.50 *W/B* ratio through the CT scan.

Figure 7 shows the pore morphology of the foam backfill material with different water–binder ratios. The pores of the foam backfill material tend to fuse with each other with the increase of the water–binder ratio. When the water–binder ratio is 0.40, the pores of the foam backfill material are uniformly distributed with a relatively uniform pore size. The boundaries between the pores are distinctly observable, showing no sign of mutual fusion between the pores.

However, when the water–binder ratio is increased to 0.50, the pore size of the foam backfill material is further increased, the boundaries separating the pores become blurred, and fusions between the large pores are observed. This trend is also reflected by the CT cross-sectional view of the corresponding samples, as shown in Figure 7c,d). The black shaded areas in the image of the foam backfill material with a W/B ratio of 0.4 exhibit a clear convergent trend when the W/B ratio is increased to 0.5. Moreover, the sample with a high W/B ratio has an insufficient paste cohesion, which makes some boundary between the pores fuzzy. 

## 4. Conclusions 

In this study, a novel self-compacting foam backfill material is developed to avoid high settlement and environmental damages caused by high filling and excavation on the back wall of the Yellow River pipeline. Based on the findings from this study, the following conclusions can be drawn:

(a) The average pore size of the foam backfill material with different density is increased with the increase of the water–cement ratio. The increments in the average size of the pores in the foam backfill material with increasing the water–binder ratio from 0.40 to 0.50 are larger than that of the samples with increasing the water–binder ratio from 0.50 to 0.60.
(b) At a lower water–binder ratio, the average size pores of the foam backfill materials and their porosity are decreased with the increase of density. On the contrary, at a higher water–binder ratio, most of the average size pores of the foam backfill material are decreased with increasing the density from 600 to 900.
(c) At a fixed water–binder ratio, the compressive strength of the foam backfill material increases with increasing the density. At a fixed density, the compressive strength of the foam backfill material first increases and then decreases with increasing the water–binder ratio. When the water–binder ratio is above 0.4, the compressive strengths of backfill materials with a density of 800 kg/m3 and 900 kg/m3 are higher than 2 MPa, which meets the strength requirements of backfill materials used for construction of the underground pipe wall of Yellow River dike.
(d) Increasing the density leads to a decrease in the diameter of air bubbles. Further analysis finds that the pore structures and the strengths of the foam backfill materials are closely related.

Overall, the developed self-compacting foam backfill material is cost-effective and holds great potential for practical applications in the underground pipe wall of big river embankment.

## Figures and Tables

**Table 1:** Performance parameters of foaming agent.

Expansion Ratio (diluted 40 times)	Bleeding Rate 1 h (%)	Sedimentation Distance 1 h (mm)
42.7	13.7%	5.7

**Table 2:** The properties and index of the test soil.

Severe γ (*kN∙m^−3^*)	Moisture Content *ω* (%)	Pore Ratio e	Plastic Limit *ω_p_* (%)	liquid Limit *ω_L_* (%)	Plasticity Index *I_P_*	Liquidity Index *I_L_*
15.8	47.4	1.28	27.2	46.7	20	0.875

**Table 3:** Mix ratios of the foam backfill material.

Specimens	Dry Density kg/m^3^	W/B %	Specimens	Dry Density kg/m^3^	W/B %	Cement kg/m^3^	FA kg/m^3^	Sand kg/m^3^	Water Reducer *wt*%
FC_600_-1	627	0.40	FC_800_-1	821	0.40	300	75	100	0.1
FC_600_-2	619	0.50	FC_800_-2	814	0.50				
FC_600_-3	621	0.60	FC_800_-3	811	0.60				
FC_700_-1	734	0.40	FC_900_-1	928	0.40				
FC_700_-2	723	0.50	FC_900_-2	916	0.50				
FC_700_-3	736	0.60	FC_900_-3	893	0.60				

**Table 4:** Fitted parameters of regression equation.

NO.	B1	B2	Intercept	R^2^
Figure 5a	−0.042	5.622	−185.624	0.99
Figure 5b	−0.076	9.333	−283.117	0.83
Figure 5c	−0.212	22.940	−650.190	0.85
Figure 5d	−3.770	403.502	−1074.992	0.97
